# A health systems approach to more effective decentralised HIV prevention: development of Malawi’s Blantyre Prevention Strategy

**DOI:** 10.1136/bmjgh-2024-016880

**Published:** 2025-02-25

**Authors:** Gift Kawalazira, Yohane Kamgwira, Sara M Allinder, Chimwemwe Mablekisi, Rose Nyirenda, Deborah Hoege, Alinafe Mbewe, Suzike Likumbo, Tyler Smith, Grace Kumwenda, Betha O Igbinosun, Charles B Holmes

**Affiliations:** 1 Blantyre District Council, Government of Malawi Ministry of Health, Lilongwe, Malawi; 2 Malawi National AIDS Commission, Lilongwe, Malawi; 3 Center for Innovation in Global Health, Georgetown University, Washington, District of Columbia, USA; 4 Government of Malawi Ministry of Health, Lilongwe, Malawi; 5 Cooper/Smith, Washington, District of Columbia, USA; 6 AIDS Vaccine Advocacy Coalition, New York, New York, USA

**Keywords:** HIV, AIDS, Public Health, Health policy, Prevention strategies

## Abstract

Achieving global targets to end the HIV/AIDS epidemic as a public health threat by 2030 and beyond requires enhanced health system capacity for HIV prevention at national and subnational levels. Specifically, this system’s capacity must enable countries to reach high-risk populations effectively, systematically engage communities to generate demand for HIV prevention services, build diverse delivery channels to meet this demand and address structural barriers that undermine prevention programmes. Integrating these capacities at the local level is especially critical to creating sustainable uptake and impact of emerging highly efficacious prevention options, such as long-acting injectable pre-exposure prophylaxis. Decentralised, locally led approaches that reflect the local context—yet are linked to national systems and policies—are needed to embed these capacities and strengthen the ability of local governments to coordinate and implement HIV prevention. Within this framework, the Government of Malawi is developing a district-based approach to enhance local institutional capacity for more effective and sustainable HIV prevention, starting in Blantyre—a large urban district noted for its high HIV incidence. This article provides the conceptual basis for, and early implementation experience of, the Blantyre Prevention Strategy (BPS), a health systems-based approach to HIV prevention that directs investments towards embedding essential functions within Blantyre City and District. The approach includes developing district-led systems and capabilities in effective disease surveillance and data-driven targeting, demand generation, quality service delivery and promoting the sustained use of HIV prevention interventions. Early learnings from BPS offer lessons for other low- and middle-income countries seeking to implement HIV prevention strategies that bolster their health system capacity and integrate with broader health responses.

**Summary box**

- The global HIV response is far behind in meeting prevention goals.
- New data-driven and locally led, integrated health systems responses to HIV prevention are needed, especially as calls to enhance country-led capacity for HIV prevention and management dominate the funding landscape.
- Malawi’s Blantyre Prevention Strategy (BPS) is a cohesive, country-led response focused on developing sustainable local capacity for effective HIV prevention.
- BPS’s comprehensive focus on building and embedding core prevention functions at the district level has built a foundation for the introduction and scale-up of injectable pre-exposure prophylaxis and has contributed to responses to COVID-19, cholera and natural disasters.
- Elements of BPS are now being replicated in the capital city of Lilongwe, and further evaluation will determine its further promise for widespread adoption.

## Introduction

 Malawi has demonstrated significant progress in combating its HIV epidemic over the last 20 years, after suffering high rates of mortality and a drop in mean life expectancy of 8 years in the late 1990s.^1^ The country has emerged as a leader in programmatic innovation and cutting-edge policymaking, from introducing Option B+ for pregnant women to access HIV treatment to expanding its treatment programme to over 920 000 people by 2022,^2 3^ contributing to Malawi’s exceptional progress towards the Joint UN Programme on HIV/AIDS (UNAIDS) 95–95–95 goals.^4 5^ Malawi has also made strides in reducing the incidence of new HIV infections, from an estimated 52 000 in 2010 to 14 000 in 2023.^6 7^ These efforts have been marked by a strong commitment to public health initiatives, partnerships with global funders, such as the Global Fund to Fight AIDS, Tuberculosis and Malaria and the US President’s Emergency Plan For AIDS Relief (PEPFAR), and have served as a testament to the potential of national leadership and collaboration.

Yet, Malawi, like many countries globally and in the region, has encountered challenges in providing HIV prevention interventions to those in need. Primary prevention interventions, from condoms to oral pre-exposure prophylaxis (PrEP) to multisectoral interventions for adolescent girls and young women (AGYW), have not yet met their public health potential in Malawi, according to UNAIDS.^8^ One of the central challenges—universally acknowledged by global HIV programmes—has been that of identifying and delivering appropriate interventions to large numbers of individuals at high risk for HIV who have little contact with the health system.^9 10^ Weaknesses in health system capacity are particularly present at the local, subnational level in districts with large and highly fragmented urban health systems, especially as they take on greater responsibility for implementing health programmes through Malawi’s decentralisation policies.

The Government of Malawi, recognising that challenges in HIV prevention could threaten long-term control of the HIV epidemic and undermine the promise of new prevention products, convened stakeholders around a new initiative in 2020. Malawi’s National AIDS Commission (NAC), Ministry of Health (MOH), Public Health Institute (PHIM) and Ministry of Local Government came together with experts to develop an innovative district-based approach focused on strengthening the local institutional capabilities needed for a more effective and sustainable HIV prevention response—including the delivery of emerging prevention products, such as long-acting injectable PrEP. The Government of Malawi selected Blantyre—the district with the highest incidence rate in the country at the time out of all its 28 districts—and pursued an approach that would leverage emerging digital technologies, support Malawi’s decentralisation policies, strengthen the institutional capabilities of its endogenous district-level health system and lay the groundwork for an HIV response more integrated with those of other health responses.

This article provides an overview of the consultative codesign process for the Blantyre Prevention Strategy (BPS), led by the Government of Malawi. It covers the conceptual framework, principles, objectives and theory of change developed through these consultations and describes its governance and early implementation.

### Blantyre: the epicentre of Malawi’s HIV epidemic

The Blantyre District Health Office (DHO), reporting to the District Commissioner, is responsible for the health and social services for the over 1.2 million people within the district, in coordination with the Blantyre City Health Office, reporting to the City Chief Executive Officer.^11^ The Director of Health and Social Services (DHSS) oversees the District Health Management Team (DHMT), which includes unit coordinators, including those focused on elements of the HIV response, that coordinate site-level staff.

Blantyre’s HIV epidemic has been challenging to control, despite substantial investment from the Malawi national government, district and partners. The district’s HIV prevalence rate in 2021 was 14.2%, nearly two times the national average, and its estimated incidence was 4.2% in 2021.^4 12^ Exacerbating factors, such as high rates of poverty and low formal employment, significantly contribute to Blantyre’s high HIV incidence.^13^ Moreover, Blantyre is Malawi’s commercial capital with robust private sector activity, which attracts frequent economic migration from other districts, including from adjacent districts with traditional sex initiation practices, with corridors of new HIV infections along roadways leading into the city from neighbouring districts and Mozambique.^14 15^

Female sex workers (FSW), men who have sex with men (MSM) and AGYW are notable risk groups, with the economic migration fueling transactional sex and high rates of sexual and gender-based violence.^16 17^

### Consultative codesign process and principles for the development of BPS

Prior to BPS, HIV prevention efforts in Malawi were fragmented and uncoordinated. Driven by the desire to address the HIV epidemic in Blantyre, partners came together through BPS, for the first time, to codesign a solution that unified numerous disparate efforts and elevated district leadership. The consultative process was guided by principles that emerged from government and civil society stakeholders (box 1); it was led by the Government of Malawi’s NAC, along with the MOH Directorate of HIV/AIDS, Sexually Transmitted Infections and Viral Hepatitis and the Blantyre City and District DHSSs. Key to this inclusive process was the emphasis on local insights and experiences in shaping the strategy through engagement with various stakeholders, including healthcare and public health professionals, community leaders and representatives from non-governmental (NGO) and academic organisations. The consultative approach led by the NAC included: (1) generative sessions among national government representatives; (2) consultations with donor and multilateral stakeholders, including UNAIDS, global fund, PEPFAR, the Clinton Health Access Initiative and implementing partners; (3) district and city-based learning sessions with district and city health management teams in Blantyre; (4) a 45-person consultation with private sector, NGOs (including organisations led by and providing services to FSW, MSM and migrants) and youth-led groups led by district and city leaders and (5) community-based organisation ‘deep dives’. This approach facilitated a deep understanding of the unique challenges and gaps within Blantyre’s health system, paving the way for a more effective and unified response to the HIV epidemic.

Box 1 **Principles for the codesign of the Blantyre Prevention Strategy initiative**

- Focus on elevating the locus of control of coordinated district and city leadership in the fight against HIV.
- Center the values, preferences and ideas of communities and front-line health workers.
- Develop HIV prevention systems that enable more effective data-driven decision-making with less operator dependence.
- Support ‘one national plan’ by linking district-based innovations with existing data systems, governance and programs at the national level.
- Leverage and coordinate resources and local organisations for greater impact.

### Conceptual framework, theory of change and BPS outcomes

**Figure 1:**
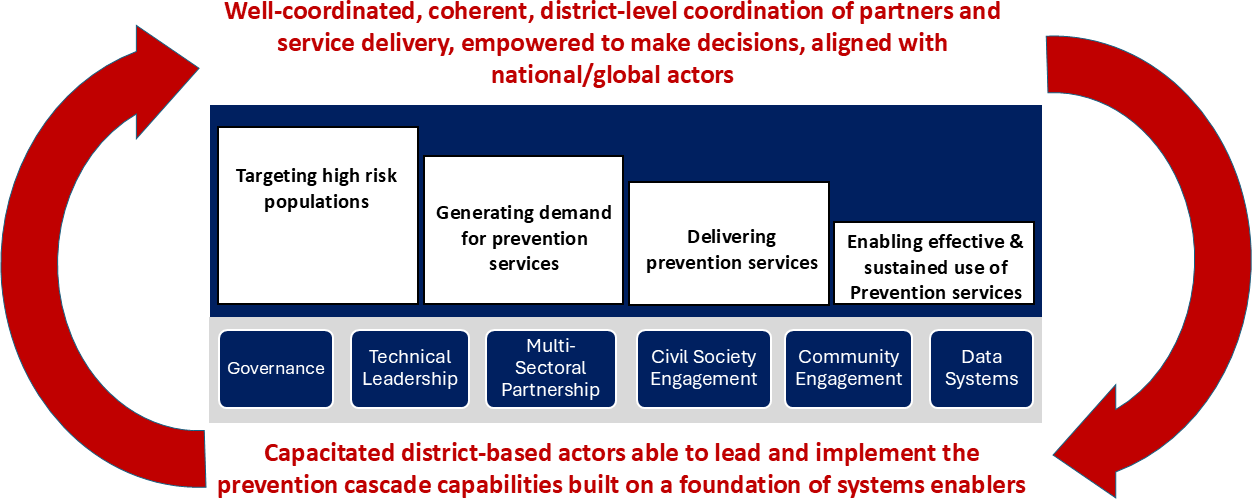
Establishing a virtuous cycle of health systems change through the establishment of core functions and key enablers of strengthened district-based prevention through BPS. BPS, Blantyre Prevention Strategy.

As Malawi’s leaders came together to define the functions needed to reinforce the health system at the district level, they selected a programmatic modification of the ‘HIV prevention cascade’ as the unifying framework for the set of integrated functions needed to strengthen the district’s capabilities for effective HIV prevention programming.^18^ These functions are: (1) targeting/reaching individuals and communities with elevated risks for HIV infection; (2) understanding community preferences and raising awareness and demand for prevention services, including new HIV prevention products; (3) delivering high-quality, accessible services along linked delivery channels responsive to community needs and (4) reducing structural impediments to sustained use of prevention products and services by those who use them (figure 1). In addition, the framework included strengthening six key health system enablers to support the performance of the functions and to facilitate their integration into the local health system.

The BPS theory of change (online supplemental annex 1) starts with a problem statement: ‘weak delivery systems have failed to reach at risk populations, leading to persistent reservoirs of infection that threaten long-term epidemic control despite existing effective prevention interventions and emerging novel HIV prevention products’. By focusing BPS on developing these functions at the district level, the government sought to centre capacity and sustainability of the HIV prevention response within local systems responsible for delivering services, with strong links to national systems. As such, the BPS theory of change included two primary outcomes: (1) development of a district-based system that will enhance the deployment and uptake of novel and existing HIV prevention interventions and products through more effective local government planning, community-driven approaches and novel innovations and (2) HIV prevention institutionalised as a cohesive, effective and sustainable country-led response, with coordinated external support.

### Implementation of BPS

#### Governance and management

The Government of Malawi defined a multilevel governance structure for BPS to operate under Malawian leadership from national to district level. This structure is overseen by a steering committee chaired by the Secretary for Health and includes national- and district-based government leaders, civil society representatives, development partners and the project funders. A project management team, including national and district government officials, was established to guide implementation, provide technical accountability and programmatic support and align the project with national priorities. At the operational level, the district and city DHSSs cochair the Blantyre coordination team to drive the execution of the project, including funded ‘consortium’ partners (local and global organisations funded to contribute technical support) and the Blantyre civil society network. A district-based coordinator with substantial government experience was hired through the NAC and seconded to report to the district DHSS, and a secretariat was established to manage daily BPS operations across partners.

#### Program elements and strategic support

Technical working groups supported the creation of tools, planning and decision-making processes and functional capabilities aligned with the prevention cascade. Each programme element was codesigned under the leadership of DHO units or city structures and linked with national systems (eg, Digital Health Division, Community Health and Health Education Services of the MOH, PHIM and NAC) to facilitate broader national adoption and sustainability.

BPS’s programmatic application of the HIV prevention cascade was meant to be intervention agnostic, meaning the technical capabilities and health systems capacities could be applied to strengthen any HIV prevention intervention. The four closely interrelated prevention cascade components include the following.

**Targeting:** A central challenge of HIV prevention is to routinely reach individuals and communities at elevated risk of HIV infection; a challenge made more difficult by the lack of timely and usable data to enhance the targeting of programme interventions.^19^ In response, BPS worked with national stakeholders to codevelop a novel, integrated data pipeline—the first that we are aware of—that pulls together data ranging from government clinical databases, PEPFAR key population partner data to census and other epidemiological information, aided by a series of data sharing agreements. A ‘front-end’ digital dashboard known as the prevention adaptive learning and management system (PALMS) was developed to allow facility and district-based staff, partners and civil society to access approved epidemiological and programme performance data from multiple sources and enable evidence-driven public health actions and delivery of targeted interventions.^20^ The dashboard format was shaped by user insights, including the results of a data user study and district- and national-level use cases. By enabling data availability and use, PALMS also serves as a backbone for other BPS functions, including an HIV-integrated disease surveillance and response (IDSR) pilot focused on building district and facility capabilities to use passive and active surveillance signals to inform targeted HIV prevention responses.

**Community engagement and demand generation:** BPS aimed to develop a sustainable solution to the decades of experience with public health interventions being initiated without adequate community engagement—often leading to poor uptake and missed opportunities for increasing acceptability.^21^ In line with the community engagement pillar of the Malawi Community Health Strategy (2017–2022),^22^ and working through the DHO’s Health Promotion Office, BPS adapted a ‘Community Lab’ model to Blantyre with local stakeholders. The Community Lab model relies on human-centred design approaches for eliciting targeted insights from communities to inform intervention implementation and demand generation activities. It was operationalised by training core district staff and partners to host focused Community Labs around key programmatic challenges identified in specific communities, such as low levels of HIV testing, or poor uptake/early attrition from oral PrEP. Community Lab-generated insights provide access to real-world motivators and barriers to health-seeking behaviour and provide a platform to collaboratively generate ideas to address them. For example, a Community Lab aimed at understanding low continuation on oral PrEP found that messages women were receiving about the timing of protection against HIV (after 3 weeks of taking oral PrEP) were being miscommunicated in a way that some clients believed they only needed to complete 3 weeks of PrEP and could stop, like their experience taking antibiotics. These insights, together with the greater availability of prevention data through BPS, enable the District Health Promotion Office, clinics and others to tailor responses and take a stronger coordination role to address health community gaps and overcome the fragmented, verticalised partner-driven health promotion landscape.

**Quality service delivery:** Quality improvement (QI) initiatives have been critical to improving HIV treatment programmes over the last two decades but have not been routinely applied to HIV prevention.^23 24^ With leadership from the MOH Quality Management Directorate, BPS codeveloped Malawi’s and the region’s first known HIV prevention-focused QI collaborative (QIC) within the Blantyre District Quality Unit. The initiative strengthened the governance and technical capacities of the quality unit while also coordinating with partners to train QI mentors and build critical capacity at facility level. District leadership decided the initial focus of the QIC would be on HIV PrEP uptake and continuation. The QIC convened multisectoral partners, including 21 public and private PrEP delivery sites and donor-funded technical partners, to establish QI performance metrics for PrEP and to facilitate sharing of data and performance across diverse partners. The QIC has become a nexus for collaboration across the HIV prevention programme, including insights from Community Labs being shared during QI coaching and QIC learning sessions to inform change ideas to improve service delivery at facilities. BPS’s multisectoral quality initiative has enabled greater coordination across implementing partners, improved connections between the DHMT and private providers and created greater health system cohesion and sustainability.

**Sustained use:** Structural drivers of HIV infection, such as poverty, gender-based violence, stigma and lack of access to healthcare facilities, can disrupt the sustained and effective use of prevention interventions. To address these drivers, BPS supported the Blantyre City Council to launch an innovative initiative to engage elected city officials and strengthen their knowledge and capacity to support HIV prevention programming. Formative research was conducted to understand gaps in the health-related knowledge of elected officials and to assess their preferences for potential structures to raise their level of engagement in the HIV response of their ward.^25^ In response, interested City Councillors were supported by a local organisation to form a ‘structural risk reduction working group’. They received training on HIV, HIV data trends in the district and their individual wards and drivers of the local HIV epidemic. Through the working group, they have discussed opportunities to better leverage the existing organisations in their wards and actions they can take that leverage their positional authority and offices to reduce structural risks in the city (eg, enhanced enforcement of local regulations). This work has now expanded to the District Council, where elected councillors have undergone an expedited version of the intervention. Recognising that the termed nature of councillor political appointments might pose a continuity challenge, discussions about further expansion to other communities and traditional structures are also ongoing.

### Use of BPS in response to COVID-19 and other public health emergencies

During the early period of the COVID-19 pandemic, district leaders sought to use BPS to extend district-based support to the COVID response through a partnership with Kamuzu University of Health Sciences. The partnership’s objectives were to expand: (1) community knowledge around COVID-19 and uptake of prevention measures in the community through the use of health surveillance assistant-supervised youth volunteers; (2) access to COVID-19 services, including triage of symptomatic cases, and onwards referral to healthcare facilities as required, through a telephone clinic based at the DHO and (3) availability of quality data on COVID-specific morbidity and mortality in the community.^26^ BPS-related improvements in governance and coordination, data use, community engagement and multisectoral partnership have also enabled strengthened coordination by the DHO in both the cyclone and cholera responses. During Cyclone Freddy in March 2023, district coordinators deployed Community Labs in displaced person camps to solicit needs and preferences for health and social services. The district also leveraged BPS-related training in passive and active surveillance through the IDSR pilot to respond to a prolonged cholera outbreak in 2022 and 2023.

### Challenges and barriers

BPS launched on 1 May 2020, concurrent with the beginning of the global COVID-19 pandemic, which delayed programme implementation. Restrictions on in-person meetings delayed local codevelopment and baseline data collection activities. Although governance structures, such as a steering committee and programme management team, were established, some members were focused on the COVID-19 response, which limited full participation until year 2 of the programme. COVID-19 also limited global technical assistance until midyear 2 due to travel limitations and inhibited a formal launch of the project locally. In addition, given its innovative nature and lack of comparators, there was some difficulty in fostering understanding of the project and its objectives. These challenges were largely resolved by the end of year 2. Other relevant challenges related to the participation of donor-funded NGO and community-based organisation partners in codevelopment and early implementation activities, as they were not initially incentivised to participate in BPS. However, as available systems support grew, partners were incentivised to participate through access to information (eg, actionable data and change ideas through the PrEP QIC) and through expanded expectations communicated by district leadership. Other challenges included the need to maintain alignment and coordination between district-level actors and national leaders, something that was attained through regular governance structure meetings and field visits. It has been helpful to frame BPS as a ‘learning environment’, which allowed testing new methods and approaches not yet embedded in national policies.

### Early effects of BPS on policy development, financing and new product introduction

BPS’s focus on building and embedding functions of HIV prevention into the health system was reflected in the Malawi National HIV Prevention Framework 2023–2027. In addition, Malawi’s NAC and MOH have supported the expansion of BPS to the next largest district in the country, Lilongwe, and built the country’s 10 000-person injectable PrEP implementation science effort, ‘PathToScale’, on the BPS platform in both Lilongwe and Blantyre. PEPFAR Malawi’s 2023 strategic direction summary noted this advancement: ‘in collaboration with government, the Government of Malawi-led BPS supported by the Gates Foundation has laid the groundwork for the scale-up of long-acting injectable Cabotegravir for HIV for PrEP and the future introduction of new products’.^27^ At a global level, the WHO’s 2022 Consolidated Guidelines on Person-Centred HIV Strategic Information recognised the BPS data pipeline and PALMS digital information system as a ‘user-defined, digital platform for holistically managing HIV programme performance’.^28^

## Conclusions and next steps

Malawi’s BPS is an ongoing programme innovation and research hub that has established a model for developing subnational capabilities for more effective and locally led HIV prevention service delivery. By establishing and embedding health systems functions within the existing structures of the district and city health teams, BPS is advancing efforts to establish more sustainable channels for investment by the government and donors. Early experiences have demonstrated the promise of building district-based leadership and capabilities, influencing policies and advancing effective PrEP use in the district. BPS stakeholders are now in discussion about the further expansion and use of the BPS model and its elements (eg, community labs and QI) to strengthen HIV prevention and injectable PrEP introduction across the country, and how the PALMS data system can support district-driven data quality assessment and surveillance of broader health and health security threats. Upcoming evaluations will provide insights into the public health functions and capabilities that have been built and embedded in district structures and the extent to which they have enabled more effective and sustainable HIV programming, including the introduction of injectable PrEP.

## supplementary material

10.1136/bmjgh-2024-016880 online supplemental file 1

## Data Availability

Data sharing not applicable as no datasets generated and/or analysed for this study.
