# Supplementary figures and images for: A health systems approach to more effective decentralised HIV prevention: development of Malawi’s Blantyre Prevention Strategy
Source: BMJ Glob Health. 2025 Feb 25;10(2):e016880. doi: 10.1136/bmjgh-2024-016880 (PMC11865780; doi:10.1136/bmjgh-2024-016880)

## Annex 1. BPS theory of change

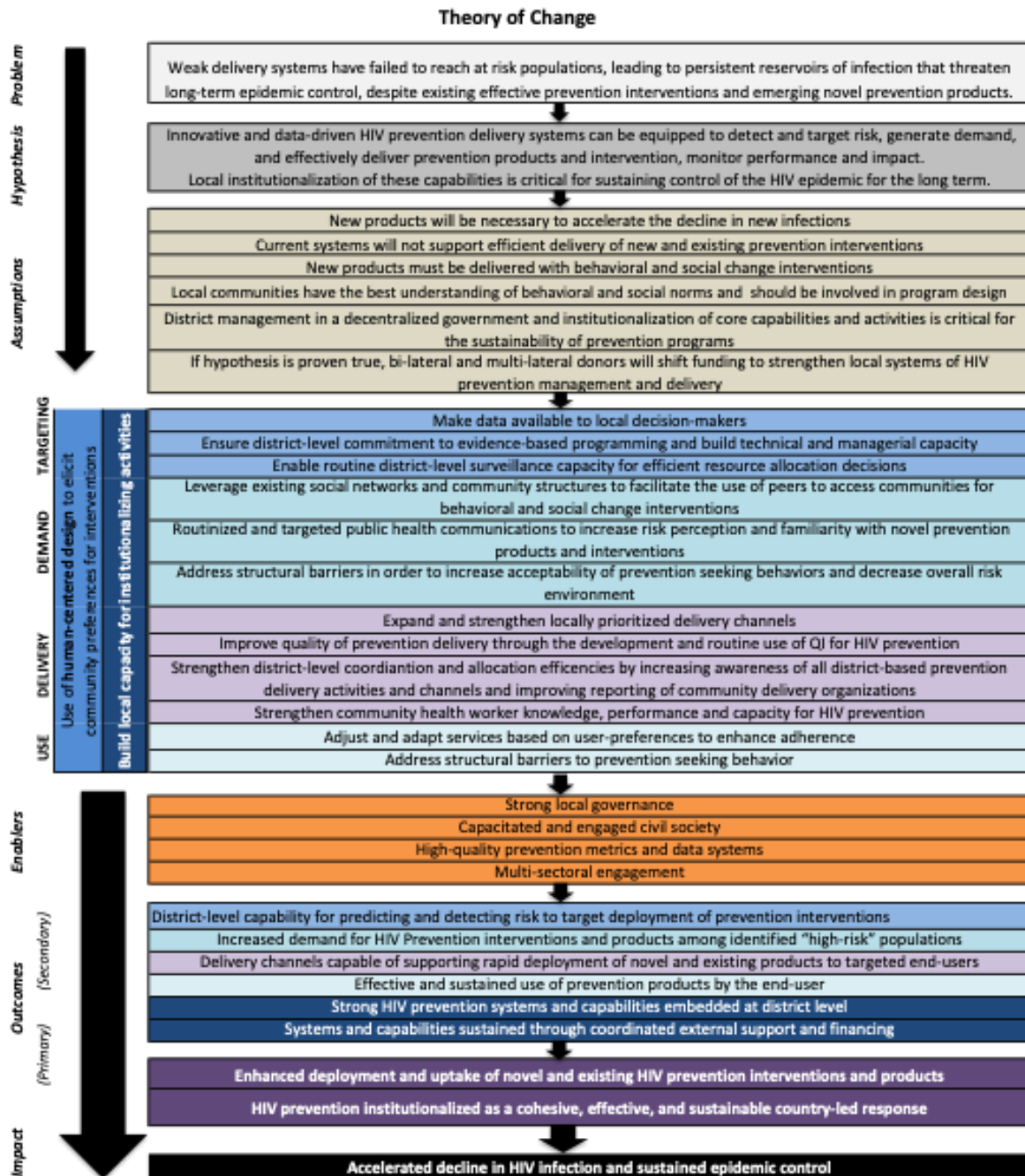

Supplement: online supplemental file 1 [file bmjgh-10-2-s001.pdf]
